# International medical leadership, collaboration and communication

**DOI:** 10.1186/s40696-016-0027-2

**Published:** 2016-11-14

**Authors:** Yael Arbel, Yehuda Zadik, Idan Nakdimon, Yuval Ran, Jacob Mendelovich, Tarif Bader, Hagay Frenkel

**Affiliations:** 1 Department of Periodontology, The Oral and Maxillofacial Center, Medical Corps, Israel Defense Forces, Tel Hashomer, Israel; 2 Department of Oral Medicine, The Oral and Maxillofacial Center, Medical Corps, Israel Defense Forces, Tel Hashomer, Israel; 3 Department of Aviation Physiology, Israeli Aerospace Center, Israeli Air Force, Tel Hashomer, Israel; 4 Medical Corps, Israel Defense Forces, Ramat Gan, Israel; 5 Surgeon General’s Headquarters, Israel Defense Forces, Ramat Gan, Israel; 6 Department of Military Medicine, Hebrew University, Jerusalem, Israel; 7 Medical Corps, Israel Defense Forces, Medical Chief Headquarters, Central Command, David Elazar St., Jerusalem, Israel

**Keywords:** Networking, Social medicine, Military leadership, Collaboration, Education

## Abstract

**Background:**

International social networking is eminent in medical practice, mainly in sharing knowledge and mutual inspiring and in social and professional bonding. Since 2006, the International Medical Course is taking place in Commander Branch at the Military Medicine Academy of the Medical Corps, Israeli Defense Forces; in which medical officers from other military forces are participating along with Israeli officers. One of the course’s objectives is international networking. The purpose of this study was to assess the level of networking in the International Medical Course compared to others means of networking, and to examine which components in the course are the most important in networking formation.

**Methods:**

Questionnaires were e-mailed to the course participants. Demographic data and data regarding the networking possibilities in the international medical course was collected.

**Results:**

The answers of 35 participants (17 Majors, 12 Lieutenant-Colonels, and 6 Colonels; mean age of 44.1 years) were included in this study. Response rate was 42%. Of the participants, 24 were Israelis and 11 from other military forces. Most of the responders (88.6%) reported the course is a major networking tool, with no influence of age, sex, rank, education profession or origin. Networking potential among participants from the same origin country was significantly higher in Israeli officers in comparison to officers from other countries (*p* = 0.001). Clinical practice and research purposes were the reason for communication in one fifth of the participants.

**Conclusions:**

The International Medical Course fulfils its purpose in forming international military medical networking.

## Background

International social networking is eminent in contemporary medical practice, mainly in sharing knowledge and experience, mutual inspiring, and collaborating in large-scale research or research which requires a large budget. Over the last decade, as the internet became a basic communication tool and with the development of social media, the global community is accessible online with much more ease than before. A survey among 24 physicians from different countries who were active users of social media revealed that the main reasons for physicians to adopt social media were staying connected with colleagues, reaching out and networking with a wider community, sharing knowledge, engaging in continued medical education, benchmarking, and branding [7].

Peer teaching engages students as teachers and is widely accepted in medical schools worldwide. Studies have shown that peer teaching has a primarily positive impact on both the peer teacher and students. Knowledge sharing was helpful in problems solving and personal mentoring of the students [1]. Among dental students networking enables the individual to develop new skills, social responsibility, team working, interpersonal communication, personal thriving and satisfaction and new occupational ambitions [2].

On the other hand however, others believe that the use of social media places doctors at a professional and ethical risk and is essentially massive time consumer of the physicians’ already busy schedules; the physicians’ priority should be treating patients rather than surfing the social media. In addition, it may be difficult to maintain a distinction between private and professional profiles [3]. Other challenges of adopting social media by physicians are maintaining confidentiality, time constrains, distrust, lack of acceptance (and support) by colleagues and employees, and information anarchy [7].

Since 2006, the International Medical Course is taking place in Commander Branch at the Military Medicine Academy; in this course, medical officers from other military forces are participating along with Israeli officers. During two weeks, the participants are familiar with Israeli military medicine, military leadership and the core values of the Israel Defense Forces (IDF)’s Medical Corps. One of the course’s objectives is the creating of an international networking. The international medical course serves as a platform for peer teaching and social interactions among participants of different ages, ranks, occupations and countries.

To best of our Knowledge, no data exists in the literature regarding networking during and following medical conferences and courses. Therefore, the purpose of this study was to assess the level of networking in the International Medical Course in comparison to others means of networking, and to examine which components in the course are the most important in networking formation.

## Methods

**Fig. 1:**
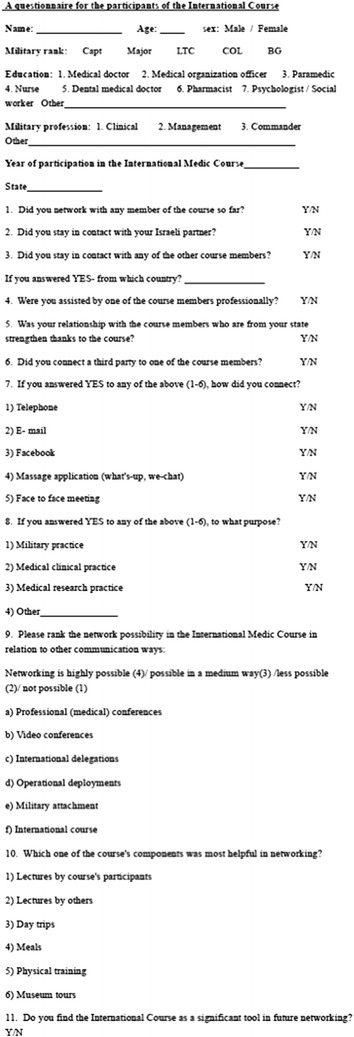
The questionnaire that was send to the course participants

Questionnaires regarding the networking in the course were developed by a committee included a variety of relevant professionals from the commanding, clinical and administrative branches of the medical corps. The questionnaires were e-mailed to the course participants (Fig. 1). A month later a second reminder was e-mailed to the participants who haven’t replied. Data was collected in an Excel-MS worksheet. For statistical analysis we used χ^2^-test for aparametric variables by PSS 22.0 software. Independent variables were profession, rank and country. A value of *p* < 0.05 was set as significant.

## Results

The answers of 35 participants (42%) were included in this study. Not all responders answered all of the questions. The demographic details of the responders are presented in Table 1. Most of the responders (88.6%) reported the course is a major tool in networking, with no influence of age, sex, rank, education profession or country.

**Table 1:** Demographic variables of participants

Variables	Israeli officers	Foreign officers	Total
N	22	13	35
Age			
Range	34–49	34–72	34–72
Average (±SD)	41.1 (±4.0)	49.0 (±11.0)	44.1 (±8.2)
Sex			
Male	15	11	26
Female	7	2	9
Military rank			
Major	16	1	17
Lieutenant colonel	4	8	12
Colonel	2	4	6
Education			
Medical doctor	11	8	19
Medical organization officer	2	4	6
Paramedic	–	1	1
Nurse	2	–	2
Dental medical doctor	4	–	4
Psychologist/social worker	2	–	2
Other	1	–	1
Military profession			
Clinical	4	4	8
Management	7	5	12
Commander	9	1	10
Combined	2	3	5

About half of the overall responders (48.6%) made a connection with one or more course participants. Responders made a connection with participants from their country at a similar rate. More of half of the responders (54.3%) have networked with another participant for professional assistant. The same proportion of responders (54.3%) has strengthened their relationships with participants from their country. About a quarter (22.9%) of the responders connected one of the course participants to a third party.

Most of the responders communicated via E-mails (81.5%). Other means of communication were telephone (51.9%), face to face meetings (40.7%), messaging applications such as WhatsApp (33.3%) and Facebook (29.6%). Messaging application was significantly more in use by responders under 42 years of age (*p* = 0.029); No other differences were found between the method of communication (E-mail, telephone, face to face meeting and Facebook) and other parameters such as age, gender, rank and profession.

Reasons for communicating between the course’s participants were military issues (54.3%), medical clinical practice or research (20%), and social networking (43.5%).

**Fig. 2:**
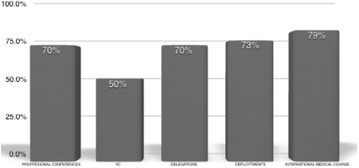
Percentage of responders on a medium to high potential of networking in different occasions

When asked about the potential to network on different occasions, 79.3% of the responders ranked the International Medical Course as medium to high potential of making of new international colleagues and networking. This is in comparison to military deployments in a foreign country (73.1%), military and/or medical delegations (70.3%), professional conferences (70.3%) and video conferences (51.4%) (Fig. 2). When asked about the Course components that offered the best platform for making of new international colleagues and networking, the responders ranked the outdoor tours (74.3%) and the meals (53.6%) as the most efficient activities; Israeli participants (16/17) ranked the tours as having a greater potential in making new colleagues and networking more than participants from other military forces (5/10; *p* = 0.008). Male participants ranked colleagues’ lectures and seminars as more effective in networking than female participants (13/20 vs. 1/8, respectively; *p* = 0.012).

Networking potential among participants from the same country was significantly higher in Israeli officers in comparison to officers from other countries (15/22 vs. 2/10, respectively; *p* = 0.011). No influence was found to age, education, gender or military rank.

## Discussion

Networking is important in the contemporary medical world. For instance, networking and cooperation among surgical teams led to a greater clinical success in the operating room; Procedures started in time, length of procedures shortened, and patient acuity decreased [4]. In addition, networking and collaboration are essential in hospital discharges which are highly vulnerable and complex events. Communication failures and problems of co-ordination resulted in delayed, poorly timed and unsafe discharges. Peer teaching and mutual inspection resulted in better outcomes for both the patients and the medical institution [5]. Le Benè and Bergus [1] have also demonstrated peer teaching to be effective; such method of teaching in medical schools worldwide benefits the learner and the teacher altogether. Research collaborations among colleagues or institutions, mentoring of senior researchers, and an ‘one on one’ training enabled a nurturing and professional research environment and problem solving in a more global manner [6].

However, in our study, clinical practice and research purposes were the reason for communication in only one fifth of the participants. It seems that the participants don’t make use of the international networking for this essential purpose. Probably the International Medical Course’s platform may not be sufficient to develop such connections. Another explanation could be that most of the participants don’t practice medicine (or medical research) as their daily job (only 22.9% practice clinical medicine), and are more likely to posse an administrative/command position.

In this study, we found that the networking potential among participants from the same country was significantly higher among Israeli officers in comparison to officers from other countries (*p* = 0.001). This finding can be explained by a ‘home advantage’ since the course is hosted in Israel and the Israelis are the most common nationality among the Course’s participants. Furthermore it is not inevitable that the Israeli officers had a previous acquaintance and the course strengthened exiting relationships.

The limitations of this article are the relatively small number of participants, and the bias that could have occurred due to the distribution method of the questionnaires, the E-mail; It is possible that the responders are more active in social media. Furthermore, the responders might be more interested in social networking than non-responders, and that was the reason they have answered the questionnaire. However despite of those limitations, the study presents a clear picture according to which a significant networking is taking place in the International Medical Course, both among participants from the same country as well as participants from different countries.

## Conclusions

Course’s participants evaluated the International Medical Course as a main tool in networking, and ranked it as a medium to high potential in networking in relation to other possibilities (delegations, deployments, video conferences, and professional conferences). Therefore, the International Medical Course fulfils its purpose in forming international military medical networking. Peer teaching is essential in networking formation; it should be emphasized on future courses. Beside military issues, propensity to medical practice and research is advised to be strengthened in order to achieve cooperation in those fields which in return would benefit the military medical caregivers and patients.

Further large scale research is warranted exploring the factors affecting international collaboration and clinical practice and research following connections made in the course.
